# The physical, emotional, social, and functional dimensions of epidermolysis bullosa. An interview study on burdens and helpful aspects from a patients’ perspective

**DOI:** 10.1186/s13023-024-03475-5

**Published:** 2025-01-06

**Authors:** Gudrun Salamon, Sophie Strobl, Marie-Stephanie Matschnig, Anja Diem

**Affiliations:** 1 HEALTH Lab, Faculty of Psychology, Sigmund Freud University, Vienna, Austria; 2 EB House Austria, Department of Dermatology and Allergology, University Hospital of the Paracelsus Medical University, Salzburg, Austria

## Abstract

**Background:**

Epidermolysis bullosa (EB) is a serious, painful, hereditary and still incurable genetic condition. Due to blistering or wounds on the skin caused by the slightest touch, a person suffering from epidermolysis bullosa is prevented from achieving the same quality of life as a healthy person. Until now, psychosocial research has focused on the description of the problems of people living with the disease.

**Objectives:**

The aim of this paper is to provide a structured overview of potential psychosocial effects of epidermolysis bullosa on the everyday lives of people with the condition and to explore helpful aspects for coping with EB.

**Methods:**

Semi-structured interviews with persons living with EB were conducted. Analyses were based on a combination of a reflexive grounded theory approach and a structured coding guide. By means of purposive sampling across three countries, a high diversity within the sample was achieved in order to obtain a wide range of possible effects.

**Results:**

A total of 17 individuals living with EB across all EB types were interviewed, resulting in 36,315 words being analysed. Psychosocial aspects of EB comprise physical, emotional, social, and functional dimensions. Identified burdens and helpful aspects in dealing with EB are described along this structure.

**Conclusions:**

Our results highlight the broad range of possible psychosocial effects caused by epidermolysis bullosa. It is particularly important to recognise those affected as individuals with their personal needs and to avoid unnecessary strains. Furthermore, emotional support is crucial in every respect.

**Supplementary Information:**

The online version contains supplementary material available at 10.1186/s13023-024-03475-5.

## Introduction

Epidermolysis bullosa (EB) is a congenital, rare and chronic condition characterised by skin fragility. Minor mechanical stress on the skin results in wounds and blisters, peelings, erosions, ulceration and scars. These occur due to structural anomalies of the skin. A lack of cohesion leads to cleavage between skin layers [1]. Depending on the level of cleavage, there are four typical types of EB: Epidermolysis bullosa simplex (EBS), junctional epidermolysis bullosa (JEB), dystrophic epidermolysis bullosa (DEB), and the especially rare type of Kindler epidermolysis bullosa (KEB). These four EB types are divided into a number of subtypes that differ, amongst other specifications, in the affected body regions, the degree of severity, the course of disease, and on a genetic level in the mutated gene(s) and the targeted protein(s) [2].

Living with epidermolysis bullosa (EB) means living with blisters and wounds and, as a consequence, with lifelong pain and itch. With adequate care [3–6], a huge part of the numerous wounds arising every day closes after some time and starts healing, but there is still no cure for the disease itself. Daily routines are influenced by painful and time-consuming wound care [7, 8] and, depending on the severity, problems with, e.g., eating and digestion [9, 10], or vision [11]. Often, these emerge in conjunction with constant caution and concern [12]. The whole life, or at least big parts of it, is affected by the disease, which makes it hard to live a close to ‘normal’ life [13–19]. Health-related quality of life can thus be considered as an essential factor to overall quality of life for persons living with EB (PEB)^1^. When the prospect of cure, and sometimes even physical improvement, are out of reach, quality of life is one of the most important measure of treatment success [20, 21]. To achieve a high quality of life, it is necessary to reach a state of wellbeing in physical, emotional, social and functional dimensions [22–24].

Prior investigations have centred on the challenges experienced by people with EB. The current study offers new empirical insights into the diversity of those challenges. Its goal is to examine the multifaceted psychosocial implications of EB, showcasing the spectrum of its potential impacts, and to identify coping strategies. Employing a combination of grounded theory and structured coding, the research presents a systematic examination of EB's psychosocial impact across four key domains: physical, emotional, social and functional aspects.

## Materials and methods

The project was given ethical approval by the Sigmund Freud University Ethics Commission (Ref: *QBGJT4EPAFIS3D87492*). Written informed consent was obtained by all participants or legal guardians of underage patients.

As this empirical research aims to focus on the lived experience of those affected by EB, semi-structured interviews were chosen as the main survey method. Based on a literature review, a semi-structured interview guide and its simplified version for underage participants were developed in a multi-professional team. In order to facilitate participation, which is crucial in research focusing on sensitive subjects [25], we offered three interview formats: a face-to-face interview, either at the EB House or at the participant's home, or an online interview via secure video telephony.

Inclusion criteria for participation in our study was a diagnosis of EB based on clinical and histopathological analysis and genetic testing. In order to assure a certain level of verbal and communication skills, further criteria were a minimum age of 10 years and German as native language. By purposive sampling, we aimed to achieve the highest possible variety with regard to age, gender, EB type, degree of severity, personal level of reflection, financial background, and place of residence (urban vs. rural). Study participants were recruited in cooperation with the EB patient organisation DEBRA Austria and the medical team of the EB House Austria [26–28].

Interviews were audiotaped, transcribed verbatim and semantically coded with the use of the software MAXQDA. We chose the reflexive grounded theory approach on the basis of its well-described aspects of reflexivity and its combination of data-driven and theory-driven methods [29]. For the interpretation of data, we combined it with a more structured approach based on thematic analysis [30, 31]. To this end, a multi-professional team developed a coding guide both deductively from the existing literature and inductively from our data collected with patients, relatives and EB experts. To reflect the diversity of psychosocial aspects of EB, it covers physical, emotional, social, and functional aspects. A total of 75 topics were identified across all interviews. The selection of topics in this paper was aimed to ensure a wide variety of different perspectives on the one hand and to select the most relevant (most frequently and extensively mentioned) aspects by quantifying the topics on the other. The sample was analysed based on the demographic data collected using descriptive statistics, including frequencies, percentages, means, medians, interquartile ranges (IQR) and standard deviations.

### Sample

A total of 21 EB patients were contacted by the medical team from the EB House Austria, of whom 17 (*n* = 17) consented to the interviews, with 47.06% (*n* = 8) conducted as face-to-face interviews and 52.94% (*n* = 9) as online interviews. The interview duration varied from 5 to 43 min, with an average of 16.31 min (IQR: 8.38, 20.28), and a word count from 673 to 5109, resulting in an average of 2136.18 words (IQR: 1013.5, 2780.0), and a total of 36,315 words (see Table 1).

**Table 1:** Overview of our 17 interview partners in regard to age, gender, EB type and interview length

Age	Gender	EB type	Interview length: time	Interview length: word count
10	male	DEB	00:07:37	901
12	female	JEB	00:12:40	2016
13	male	JEB	00:08:38	912
17	male	DEB	00:08:37	1011
21	female	DEB	00:24:34	2903
23	male	EBS	00:14:45	2399
23	male	DEB	00:05:09	673
25	male	EBS	00:16:21	1554
26	female	DEB	00:33:40	4753
27	female	EBS	00:13:50	1387
28	male	DEB	00:12:25	1795
30	female	DEB	00:15:21	2054
30	female	DEB	00:42:51	5109
33	female	DEB	00:16:07	2657
41	female	JEB	00:07:10	1016
42	male	JEB	00:13:58	1592
49	male	EBS	00:27:08	3583

The average age of the PEB was 26.47 years (MD = 26.00) ranging from 10 to 49 years. Gender was balanced with 47.1% being female and 52.9% male. The sample consists of all types of EB, with the exception of Kindler EB: EBS and JEB were equally represented with 23.5%, while the majority of participants (52.9%) were diagnosed with DEB. The degree of severity was rated by an EB specialist as followed: 29.4% as mild, 29.4% as moderate and 41.2% as severe. Most participants had been diagnosed within the first six months of their life, with only two PEB being diagnosed later than the first year. Two had another family member with a diagnosis of EB. At the time of the survey, 23.5% of the PEB indicated that they were currently in an acute difficult EB phase. All participants were native German speakers, with 64.7% participants from Austria, 29.4% from Germany and 5.9% from Italy (see Table 2).

**Table 2:** Sociodemographic description of the study sample

Sample size	17 Persons living with EB		
Average age	26.47 (SD = 10.72; MD = 26.00) Range from 10 to 49 years		
		Participants (n)	Percent (%)
Gender	female	8	47.1
	male	9	52.9
Place of residence	Austria	11	64.7
	Germany	4	23.5
	Italy	2	11.8
EB type	EBS	4	23.5
	JEB	4	23.5
	DEB	9	52.9
	KEB	0	0
EB severity (EB specialist assessment)	mild	5	29.4
	medium	5	29.4
	severe	7	41.2
First EB diagnosis	at birth	5	29.4
	within the first 6 months	7	41.2
	6 months to 1 year	3	17.6
	more than a year	2	11.8
Last challenging phase in regard to EB	at the moment (acute)	4	23.5
	less than 6 months ago	6	35.3
	6 months to 1 year ago	1	5.9
	more than 1 year ago	5	29.4
	could not estimate it	1	5.9

## Results: Disease burden of people living with epidermolysis bullosa and helpful aspects

### Physical aspects

#### Pain, itch, wound care, smell

Physical pain is a constant companion of PEB. Itching and severe pain were often mentioned in the interviews as a source of negative emotions. Wound care is a burden in itself. Dressing changes, bathing and further care are described as painful, uncomfortable and annoying. Furthermore, open wounds can produce an unpleasant odour, which can be bothersome for those affected themselves and cause feelings of shame when interacting with others. *“The smell and the re-doing of bandages next to them, of course, that wasn't cool for the others either. […] I actually don't think that they deliberately ostracised me for that.”* *(B15)*^2^

#### Motor skills and mobility

Mobility and motor skills are restricted when feet and hands are affected by blisters and wounds. In addition, PEB often report that they get tired during long journeys, making hikes or long walks impossible. There are also PEB who rely on mobility aids such as electric wheelchairs. As a consequence, spontaneity is often impaired due to mobility. *“Because I can't say now ‘I'm going there quickly by train’, but you always need a preliminary procedure, need to register and so on, and I am very limited.” (B9)* The execution of fine motor tasks also requires more patience, as those affected often need to ask for help with everyday things.

#### Sports

The subject of sport is addressed remarkably often by the interview partners, especially by those with a mild or moderate degree of severity. The restrictions in sport seem to have a huge impact, both personally and as a facilitating environment for building social contact. *“It actually has the most influence on my life that I simply cannot participate in many sports. […] I can't ride my bike normally, either. I need a bike with three wheels. And with an electric drive.”* (B3) For some, it is difficult to accept that the disease is limiting them. *“I'd actually like to do that, and that sounds cool, but I just can't do that. That's where it is difficult to accept.”* (B1).

#### Nutrition

Dietary restrictions can also be very stressful when certain foods that would delay wound healing or solid foods in general have to be avoided. For many, eating takes longer. One answer to what is particularly difficult in dealing with EB was, *“Food. Yes, eating.”* (B17) This shows how paramount this burden can be. When the gastrointestinal tract is affected by EB, acute blisters in the oesophagus are associated with swallowing difficulties and severe pain. *“Not being able to drink anything is of course a threat to one's existence.”* (B7).

#### Helpful aspects

**Mobility aids** help to enhance the quality of life by restoring a certain degree of autonomy. Due to the fragile skin, electronic wheelchairs are preferred. If possible, **sports** or exercise in **nature** are experienced as a helpful resource. Concerning health care, one of the most important helpful factors is that PEB receive the **right treatment** for their condition. Hence, there is a need for **EB experts** in the medical field (doctors, nurses, professional carers, and other specialists). It is further considered very helpful to have an expertise centre helping with any medical issue related to EB, like the EB House in Austria. In addition, easy-to-reach telephone or e-mail support for acute enquiries and a short response time were described as helpful. Patients would like their doctors to be **honest** about diagnoses, prognoses and, above all, about what they should prepare for. Furthermore, patients want doctors to be honest about their own knowledge of the disease. *“A successful treatment is, first, that the doctor knows EB. Or, if he doesn't know it, that he tells me so.” (B1)* Regular **medical check-ups** can reduce the legitimate fear of skin cancer. While some PEB do not want to change a medical treatment or bandages that work for them, others are very open to new suggestions. “*For me, a successful treatment is certainly one that manages to make daily life a little easier, to relieve the pain and perhaps to get information about innovations in research, about new applications that exist.” (B7).*

### Emotional aspects

#### Limitations in life

In the psychosocial field, one of the greatest limitations seems to be the struggle to live a ‘normal’ life. *“I must admit that sometimes I struggle because I would love to do some of the things that others do.” (B15)* It is described as frustrating that every beloved activity comes with the need for evaluating the risks of causing subsequent physical discomfort. *“That there are a lot of things that I can't do and the others can. That's stupid that I often can't do things for too long that I actually enjoy […] because then my feet will hurt.” (B5)* EB affects everyday life even in aspects such as the individual appearance, which is determined by the need to choose clothing with regard to the risk of possible friction. “*Basically in all areas somehow. Starting with which clothes and which shoes I can wear.” (B2).*

#### Individual course of disease

EB has many different forms, and even within a given type, there are huge differences in the degree of severity as well as in the course of the disease. It is hence especially important to point out that not only every single person affected by EB is individual, but also their illness itself, which requires a highly personalised healthcare plan. It is consequently particularly difficult for those affected to obtain clarity and certainty about their condition. *“What makes it difficult is that it's different for everyone affected, which means they can't say that always helps with that, it's a bit of a gradual approach and everyone has to find what works for themselves.” (B2)* This leads to insecurity within PEB. *“Then you go back to the doctor, and then they say, oh actually, this ointment might be much better, and then I always have the feeling that I don't know the exact right thing yet and that I could keep asking again and again.” (B1)* In addition, the course of the disease is unpredictable and can vary greatly from day to day, which severely affects the ability to plan ahead and organise one’s life. *“What's difficult is that it’s unpredictable. You can do well today, then something happens to you and you can't walk for a week, for example.” (B2).*

#### Dependence

Depending on other people is described as another psychological burden. The need for support can arise from reduced mobility or fine motor skills, as well as for wound care in affected body regions that cannot be reached by PEB themselves. When asked what was difficult in dealing with EB, one interview partner replied, *“That you are always dependent on the parents. And that you are so dependent on other people. At 21, most others are independent […]. And I'm still sitting at home, and I need help with every little thing.” (B16).*

#### Constant confrontation

The constant confrontation with the disease makes it hard getting EB time-offs. PEB find it difficult to be seen primarily as someone affected by an illness and that every conversation is overshadowed by EB. *“It's just about the disease they're talking about. And that's not really what my life is.” (B15)* Another burden associated with the rarity of the disease is the recurrent search for causes. PEB often report feelings of depression when faced with the question of why they have EB. *“Sometimes I just sit down and ask myself why I have EB.” (B12).*

#### Helpful aspects

A **positive attitude** can be of great support in emotionally coping with the disease. *“And that's why I’m not working myself up and try instead to see the situation as it is, it's like that, so approach life positively, see the good.” (B14)* Participants express that quality of life can be enhanced by finding **fulfilment**, whether in hobbies, family or something else. Several interview partners also mentioned religion or spirituality as such a source. **Acceptance** plays an important role in order to focus on the factors that can be changed in one’s life. *“Well, you learn to live with it. Because at some point you realize that it's a disease that, in fact, you're always going to have. This cannot be changed. I can only change my attitude towards it.” (B7)* When it comes to unchangeable aspects, it can sometimes be helpful to consider workarounds and **alternative solutions**. *"I tried never to let it affect me, but to always find my way. If one thing didn't work, I’ve found a way around it. Yes, I actually feel strong doing so." (B2)* Being able to do what healthy people do can give PEB a sense of living **a life as close to ‘normal’ as possible**. *“I just keep noticing that the most beautiful thing for me is the normal life, being able to do everything with friends, experiencing beautiful things. For that, you sometimes take the risk of one or another wound, that's part of it.” (B1)* **Taking care of oneself** contributes to a feeling of control and independence. *“Because I can do a lot myself anyway, in the evening I can take care of my wounds myself, and if that was not possible anymore, that would be a big challenge.” (B9)* When dealing with pain and itch, distraction can help to think of something else. **Distraction** is the most frequently mentioned helpful factor by all interview partners. While adults pursue their hobbies or focus on their family, children distract themselves by playing or using electronic devices. *“Distraction. This is the best medicine, and distraction has thousands of facets. It may be that today is a TV day, then television helps. Music helps, friends help, spending time with my child helps.” (B14).*

### Social aspects

#### Visibility

The almost inevitable visibility of the disease and the reactions of others to it are often perceived as a burden by PEB. *“You can see it right away, of course you worry when you're somewhere, in summer, in the swimming pool or by the sea. Of course, even when you meet people. I think that's a huge point.” (B2)* Some try to hide the affected parts of their skin, which in turn leads to restrictions in everyday life, like not being able to go swimming or always wearing long sleeves. The problem of visibility is also an issue in relationships. *“It influences my relationship, for example, I do not feel completely comfortable with always showing all parts of my body.” (B1).*

#### Reactions of others

People's reactions to disease-related appearance are very diverse. During adolescence, especially at school, they are often quite negative. *“And during childhood, you are in school and kindergarten and you get teased. And you can't avoid them.” (B14)* Due to a lack of knowledge, PEB may be perceived as disgusting or contagious, which leads to social exclusion and a consequent lack of social contact. *“It's more likely that the others keep a certain distance as long as they don't know what it is.” (B6)* Some children are even cruel out of curiosity. *“And yes, there actually was one boy in primary school who threw me to the ground just to see how fast it [the skin] really rips open.” (B15)* In addition, the constant questions of people who have no knowledge about the disease are perceived as uncomfortable and annoying by those affected. *“Well, at school, of course, it's not always pleasant with your peers who say ‘What’s wrong with your skin?’. They stare at you. I have to admit that's causing mental stress.” (B7).*

#### Medical encounters

Medical encounters with doctors not specifically trained for EB were described as a challenge. This is particularly problematic when immediate care is required for acute injuries. In one case, a severely wounded PEB had to explain to the doctor what to do. *“Well, he was a bit overwhelmed. I mean, I told him what to do.” (B15)* Unsuccessful treatments can cause physical and emotional distress. *“Back then we went to a lot of doctors who gave really weird advice. I always used to be quite skinny, so there were some suggestions of some absurd cures and diets and other stuff.”* (B7) EB also needs to be considered in treatments that may seem unrelated at first glance, such as going to the dentist or the gynaecologist, which can otherwise cause great pain.

#### Helpful aspects

In general, it helps to have a **supportive environment**. *“Family, friends you can talk to, laugh with. That's the biggest support.” (B6)* In addition to family and friends, also **animals** are mentioned as helpful companions. The disease should not be the focus of social interaction. PEB simply would like to **be treated normally** by others. *“And then, when there are a handful of people left who really talk to you, who understand that and who totally want to see you and don't think of you as of ‘the poor girl’, but instead that this is normal. That's what's called friendship.” (B10)* Social interaction is much easier with people familiar with the condition. *“That's definitely nice when you realize that there are just people who have heard about it before and have no fear of contact.” (B1)* It is important to **make the disease visible** and understandable to others in school classes, workplaces, etc. PEB report that their quality of life has improved since they stopped hiding their condition and became more open with their environment. *"Actually better. More open in contact. I used to want to show myself less because of my skin. But now I do and it feels good." (B4)* Also, PEB want to decide when and how they want to be confronted with the disease. **Psychological or psychotherapeutic support** and **rehabilitation stays** have the potential to improve quality of life, but require intrinsic motivation. The **exchange** with other people in the same situation is considered as very helpful, too. Support groups or private meetings of PEB pool a lot of experience and expertise. *“So I go there when I want to or when I feel the need. The meetings themselves are good for the exchange of experience and everything.” (B15).*

### Functional aspects

#### Organisation

EB requires a lot of time and organisation. The strict daily medical schedule is very time consuming and therefore other activities may not be possible due to this time constraint. “*Very often, I have moments when I would like to be outside and play, but I can’t, because I have to change my bandages or take care of my wounds.” (B12)* As soon as the skin is put in second place, the physical condition suffers severely. Furthermore, any special activity needs to be planned in advance to ensure the appropriate medical care, which then as a consequence reduces the possibility of spontaneous activities. *“Planning is difficult. Often you can really only see it step by step, day by day.” (B14).*

#### Financial effects

Medications, special treatments and dressing materials are expensive and are not fully covered by the health insurance. *“If I treated my wounds the way I should, it would be a financial burden.” (B6)* Nonetheless, adequate and sufficient wound care supply is directly linked to the quality of life. *“I sometimes get annoyed when things like this aren't and can’t be taken over by the insurance when it's actually clear that I just need this for my skin care, for example bath supplements or creams, stuff I couldn't live without or would, at least, not have a good quality of life.” (B14)* Other medical aids, such as a wheelchair, are provided by the health authorities only after a complicated application procedure. Similarly, applications for domestic help or personal assistance are perceived as excessively complex and burdensome procedures. *“It is a steeplechase at these administrative offices.” (B15).*

#### Helpful aspects

**Financial support** for consultations with specialists, dressing material, mobility aids, etc., covered by public or private institutions, is very important for PEB. Most of these can only be requested with a high level of knowledge about the **application process**. In this respect, the help of trained social workers is often mentioned as a great support. Regular **domestic help** is experienced as a relief. *“That helps me a lot that someone comes to my home once a week and gets stuff done.” (B15)* **Professional assistance** can allow the main care takers to get some time off, which is also described as a relief for the whole family system.

## Discussion

The detailed information obtained from 17 PEB provides an in-depth insight into individual perceptions. In total, 75 topics addressed in the interviews were categorised into four main themes: physical, emotional, social and functional aspects. The diverse physical effects of EB have already been described in detail in previous literature [1, 2]. However, the psychosocial aspects and their impact on the lived experience of those affected are mostly presented as mere ‘accompanying factors’. Although these latter factors are often caused by physical symptoms, their recurrent mention in the interviews highlights their significance and indicates that they themselves exert a considerable influence on the experience of living with EB. It is therefore imperative to consider these factors individually. Concerning the described burdens, our findings are consistent with previous qualitative research [7, 10, 13, 15–18], but offer a more systematic and comprehensive overview of the topics across all degrees of severity of EB. While previous research has mainly focused on the problems of PEB, this paper adds empirical data on what is considered as helpful in dealing with these burdens [32].

The topics of the first category **physical aspects** have already widely been highlighted by various medical studies. Similarly to our findings, pain, itch and wound care are described as key issues of EB [7, 8]. Restrictions in motor skills and mobility can be caused by acute or chronic wounds or, in some types of EB such as RDEB, by degenerative mitten formation of the fingers and feet, and were also reported by our participants [33, 34]. In addition, the discomfort and distress caused by an EB-affected gastrointestinal tract have been identified as significant issues that require medical attention, but also have major psychological and social implications [10, 35, 36]. Despite these diverse physical limitations, sport is often emphasised by participants as a relevant topic. In particular, people with milder forms of EB often mention it in relation to social contexts, whereas people with moderate forms of EB, and therefore more severe limitations and physical strain, emphasise the limitations. The link between physical symptoms and their psychosocial consequences therefore seems to depend on the degree of severity and should be examined in more detail in future studies.

Assistive aids, such as mobility aids, electronic wheelchairs or occupational therapy methods, allow for a higher degree of mobility and hence participation [37]. Furthermore, access to regular medical examinations was emphasised as a helpful resource for EB. We found that the increase in information about EB and its prognosis might cause anxiety and negative emotions, but that a realistic assessment can also enable a realistic preparation for the future and can lead to a feeling of control in periods of uncertainty. Antonovsky already emphasised the importance of comprehensibility as the basis for a sense of coherence and thus the ability to counteract stressors and establish resources [38]. Consequently, a realistic assessment and comprehensibility have a major influence on the availability of resources and the ability to deal with burdens. An open and honest exchange with doctors regarding diagnosis and prognosis was repeatedly identified by the participants as both desirable and helpful. Even with knowledge of a diagnosed EB subclassification, the individual presentation must always be taken into account, which requires an individualised medical approach.

Concerning **emotional aspects**, the desire for a ‘normal life’ was found for all degrees of severity. This has already been shown to be especially challenging for persons with a rare condition [39]. The individual assessment of one's own situation is based on the constant differentiation from other individuals who are not affected [40]. The limitations specific to EB are thus made explicit and emphasised, which can often lead to questions about the meaning of the condition and may be accompanied by negative emotions or depressive feelings. The occurrence of depression and anxiety in EB is in line with previous studies [12, 41]. Furthermore, the understanding of health and illness can be decisive for how PEB see themselves and to what extent they perceive limitations and resources. Quality of life as a construct can be used in this context, as it reflects a person’s individual view of their situation and therefore also includes the understanding of illness and health. Numerous studies have shown that EB has a significant influence on quality of life [24, 42–44]. However, there is a huge diversity within the EB population, and quantitative approaches often fall short in this respect. This is reflected in the repeated emphasis by PEB on the individuality of the disease. The unpredictability of individual disease courses makes it particularly difficult for PEB to gain any kind of certainty about their condition and future, leading to feelings of uncertainty and loss of control. The need for assistance (sometimes lifelong) with activities of daily living and wound care further complicates the situation, leading to feelings of dependency [19]. This can lead to conflict as the person ages, and can ultimately become a burden through feeling like a burden. More research focusing on the influence of EB on the transition from adolescence to adulthood is needed. One point that has not been mentioned in previous studies is that the perceived quality of life of PEB is higher when finding fulfilment, be it in for instance hobbies, religion and spirituality.

While **social** connections can be a strong support, social circumstances, particularly the reactions of others due to the visibility of EB, are also highlighted as a possible burden. It is thus not only the disease itself, but also the reactions of others that PEB have to cope with, which is already not easy for adults, but perhaps even more so for children and adolescents [13, 15, 45]. In our results, there were more examples and expressions of such situations in the retrospective accounts of childhood. Therefore, there may be a possible difference in perceived burden according to age. One possible reason could be that adult PEB have already acquired better coping mechanisms over the years, such as setting boundaries and selective communication with the environment, which has been described in other rare diseases [46]. In this context, the need for education about the disease becomes apparent: Participants emphasise that it is important for the general public to know about EB and that it helps to be treated as a normal person. Stigma, exclusion and being stared at are issues that people with EB face on a daily basis, and have been shown to highly impact quality of life and mental health [15, 47]. Some PEB report that their quality of life has improved since they stopped hiding their condition, although this can be a burden in itself. The visibility of the condition also affects intimate aspects of relationships, such as romantic and sexual relationships. Similar to previous studies, the interaction with family and friends has been described as affected by EB [24]. At the same time, many experience the relationship to family and friends as mostly helpful in our study. Social contact with other PEB was also mostly described as helpful, as was the sharing of information about treatment and coping strategies.

Medical encounters were also highlighted as a relevant topic in the context of social aspects. In contrast to the literature, in our sample the majority of children with EB had been diagnosed at a very young age of a few weeks or at least within the first year of life [48]. However, there is still a lack of knowledge about EB among medical staff outside of EB centres of expertise, and doctors from specialities not directly affected by EB should take the disease and its pain into account in their treatment. Especially in emergencies, when PEB are in a vulnerable situation, social norms can get in the way and create an imbalance between uninformed doctors who decide against the knowledge of expert patients [49]. In such contexts, PEB emphasized openness, honesty and equal communicating as helpful. Psychological or psychotherapeutic support and rehabilitation stays have the potential to improve the quality of life of PEB, but require intrinsic motivation as well as time, financial and organisational effort [50].

The **organisational** and **financial** burden due to EB was highlighted as a relevant topic by our participants and is also reflected in existing literature [51]. PEB often have to plan their time and resources around EB and daily wound care. Leisure activities usually have to come second, with the unpredictability of the disease adding to the burden. These organisational and time restrictions also mean that opportunities for social interaction and friendship are missed [52]. The participants emphasized that regular domestic help and professional assistance can be a relief for the whole family [53]. As wound care often requires a great deal of specialist EB knowledge, which even professionals often lack, home carers usually need to be trained extensively by the parents in the beginning. Although adequate and sufficient wound care is perceived as a key factor to quality of life, the costs are often not sufficiently covered by the health care system [50, 54]. Obtaining funding often involves time-consuming application processes that require a great deal of expertise and organisational talent. In a daily life where time is already reduced by EB, such demands for financial support are a particular challenge and burden, not only for the PEB themselves, but often for their whole family. In this context, professional support at eye level, e.g., by social workers, could help to reduce the organisational burden.

Finally, it is important to consider that the four categories and their subtopics are all interrelated and interdependent. Improving one aspect could in turn have a positive effect on another one and vice versa. Most participants told us that their dealings with EB have changed over the years. Many have become more accepting, which also goes hand in hand with a greater sense of normality. As people get older, their self-image changes, which is often accompanied by greater openness and sociability. Greater autonomy also leads to an experience of greater self-efficacy. Future studies should examine such interdependencies and how to improve the quality of life in EB in the long term. To date, however, it is essential that all four areas are taken into account individually in the daily lives of families, in clinical practice and in research in order to consider life with EB in all its diversity.

## Critical reflection

Regarding the sample and its characteristics, the sample size is high given the rarity of the disease and the qualitative research method. Female and male patients were equally represented in our study. The age range of 10–49 years allows to capture the diversity of the effects of EB in as many different life situations as possible. Given that a higher degree of severity is associated with a significantly shorter life expectancy, this age range is high [55]. The perspective of children with EB is only included in retrospective explanations of the participating PEB, which could lead to a potential bias. Our purposive sampling strategy aimed to target the full range of disease severity rather than EB type, as there are large differences in the individual expression of EB between different EB types and subtypes, and even within these types. The degree of severity is used as a proxy in this regard. In doing so, the broad range of burdens has been put at the centre of attention instead of the classification into EB types and their typical symptoms. This strategy makes it possible to map the psychosocial effects to a greater extent, but naturally, not every effect described above affects every EB patient. The family system is currently only analysed from the patient's perspective. It is likely that different perspectives will emerge, at least in part, if the views of relatives are also explored. By including patients from three different countries, country-specific cultural and systematic differences, e.g. resulting from different healthcare systems, are reduced and reflect the situation in countries with good public healthcare. Even though we aimed for a high diversity within our sample, further investigation in regards to the worldwide EB population is needed. In order to consider the influence of systemic problems due to differing health care systems, further country-specific studies should be conducted, such as those in, e.g., Austria, Ireland and Spain [45, 50, 56]. Based on this, advantages and disadvantages of the respective system could be derived and possible implications transferred to other countries. A next step would be to investigate the identified topics on a quantitative level with a larger EB population, ideally on a multi-national level.

Regarding the methodology, the somewhat unusual combination of grounded theory and thematic analysis makes it possible on the one hand to identify previously unknown or little-described aspects of the disease and associated experiences, and on the other hand to bring these into a systematic structure. Of course, reducing individual dimensions of experience to central recurring themes involves a necessary simplification and potentially selective summarisation of the findings. For this reason, it is important at this point to underline the wide variation in the lived experiences of people with EB. Nevertheless, certain themes permeate the individual experience, which will be examined for their implications in the next section.

## Implications

The burdens to be taken care of include financial aspects as well as reduced mobility. Wheelchairs should be financed by the health authorities and public transport should be adapted. Assistance, if necessary and wanted, should also be available for everyday life. Such help can reduce the burden that PEB face in everyday life. However, many of the stresses and strains are unavoidable and lead to negative emotional effects. It is therefore important to offer support in all areas and to find helpful factors in order to strengthen PEB.

Many PEB emphasise that they are proud of their independence and can do a lot themselves. Taking care of oneself can contribute to a feeling of control and independence. However, many of the daily tasks and needs require assistance by others in order to live a self-determined life. On a personal level, it is particularly important to provide emotional, medical, informational and instrumental support to those affected. This especially includes eliminating difficulties in life that are not necessary. External assistance, whether it be for wound care or personal assistance, not only reduces the burden on the caretakers (mostly family), but also relieves the children or young adults affected as it reduces the feeling of guilt. Support groups as well as other professional support, especially psychological support, is described as helpful by many, but only if accessed on a voluntary basis.

Several of our interview partners talked about their concepts of a deeper meaning of life, either through spirituality or religion or by finding something fulfilling. For them, regarding their disease as something meaningful as well as having a positive attitude towards life, helps in coping with it.

It is essential for PEB to be taken seriously and to be seen and treated as human beings and not just as ‘sufferers’ and ‘patients’. It is helpful to share experiences with people who are in the same situation. However, this does not imply that the same things might help everyone. Although the disease is the same, people differ in terms of their needs. What works for one person, does not necessarily work for another, hence individual solutions need to be found, again and again. In this concern, it helps to consider PEB and their relatives as experts for their own case, and to pool individual and medical expertise for finding the best solution for the individual concerned.

## Conclusion

Epidermolysis bullosa is a disease that is associated with a wide variety of individual clinical pictures and manifestations. This physical diversity is accompanied by a high variance of different psychosocial and financial factors that are affected by EB. In order to accommodate this diversity, but also to create a concise overview of all relevant topics, a purposive sampling strategy was combined with the qualitative methods of grounded theory and thematic analysis. The results showed four main areas that can be affected by EB: physical, emotional, social, and functional aspects. This study was designed to serve as a thematic map to identify relevant EB topics that require more in-depth consideration. It thus provides a systematic overview of the major impacts of EB that should be consistently taken into account in future clinical and psychosocial research, as well as in everyday medical practice.

## Supplementary Information

Additional file 1 Interview Guide

## Data Availability

The data generated during and/or analysed during the current study are not publicly available nor are they available on request due to the rarity of EB, which limits anonymity even with pseudonymisation or exclusion of personal data.

## Footnotes

1 We decided not to use the term ‘EB patient’ widely used in literature as we want to address all aspects of a person living with EB instead of reducing the person to his or her medical symptoms and needs. Instead, we will use the abbreviation PEB for ‚person(s) living with EB‘.

2 Direct quotes from interviews were translated from German into English by the authors.
