# Supplementary material for: The physical, emotional, social, and functional dimensions of epidermolysis bullosa. An interview study on burdens and helpful aspects from a patients’ perspective
Source: Orphanet J Rare Dis. 2025 Jan 6;20:3. doi: 10.1186/s13023-024-03475-5 (PMC11705948; doi:10.1186/s13023-024-03475-5)
Supplement: Supplementary file 1 — Additional file 1 Interview Guide [file 13023_2024_3475_MOESM1_ESM.pdf]

Interview Guide

	MAIN QUESTIONS	PROBING QUESTIONS
1	How does EB affect your life?	*How does EB affect your leisure time?*<br>*How does EB affect your work?*<br>*How does EB affect your partnership?*
2	How did your experience of EB change over time?	
3	How do people around you deal with your EB?	*How does your family deal with EB?*<br>*How do your friends deal with EB?*<br>*How do other people around you deal with EB?*
4	What is especially difficult in dealing with EB?	*Medical aspects*<br>*Psychological aspects*<br>*Social aspects*<br>*Financial aspects*<br>} *How do you deal with that?*
5	What do you experience as helpful in dealing with EB?	*What helps you when you are not feeling well?*
6	There is quite a wide range of support for EB, e.g., medical, social, and psychological support. Where do you turn to in which matter?<br><br>Are there any other persons you are turning to?	*Where do you turn to for medical support?*<br>*Where do you turn to for psychological support?*<br>*Where do you turn to for social support?*<br>*Where do you turn to for financial support?*
7	What do you experience as especially helpful in this case?	*Medical support*<br>*Psychological support*<br>*Social support (Personal network and „EB Community")*
8	What do you think makes a treatment successful?	
9	What sort of support would you wish to have that, until now, is not or not sufficiently available?	
10	Would you like to add something?	

Thank you very much for your precious time and your answers.
